# A novel homozygous missense variant in *ARSK* causes MPS X, a new subtype of mucopolysaccharidosis

**DOI:** 10.1016/j.gendis.2023.06.003

**Published:** 2023-07-10

**Authors:** Miao Sun, Cornelia K. Kaminsky, Philip Deppe, Mai-Britt Ilse, Frédéric M. Vaz, Barbara Plecko, Torben Lübke, Linda M. Randolph

**Affiliations:** a Division of Genomic Medicine, Department of Pathology and Laboratory Medicine, Children's Hospital Los Angeles/Keck School of Medicine of USC, Los Angeles, CA 90027, USA; b Department of Radiology, Children's Hospital Los Angeles/Keck School of Medicine of USC, Los Angeles, CA 90027, USA; c Department of Chemistry, Biochemistry, Bielefeld University, Bielefeld 33615, Germany; d Amsterdam UMC Location University of Amsterdam, Department of Clinical Chemistry and Pediatrics, Laboratory Genetic Metabolic Diseases, Emma Children's Hospital, Meibergdreef 9, Amsterdam 1100 DE, the Netherlands; e Amsterdam Gastroenterology Endocrinology Metabolism, Inborn Errors of Metabolism, Amsterdam 1105 BK, the Netherlands; f Core Facility Metabolomics, Amsterdam UMC Location University of Amsterdam, Amsterdam 1100 DD, the Netherlands; g Department of Pediatrics, Division of General Pediatrics, Medical University of Graz, Graz 8036, Austria; h Division of Medical Genetics, Department of Pediatrics, Children's Hospital Los Angeles/Keck School of Medicine of USC, Los Angeles, CA 90027, USA

Mucopolysaccharidoses (MPS) are a group of rare inborn errors of metabolism caused by defective lysosomal enzymes which prevent cells from degrading and recycling certain carbohydrates and fats, resulting in the storage of glycosaminoglycans in cells throughout the body. This leads to multisystem abnormalities involving bone, connective tissues, brain, blood, spinal cord, skin, and other tissues. In humans, seven distinct types and many subgroups of MPS have been classified, and each is linked to deficiencies of the specific enzymes. Very recently, two studies^1^^,^^2^ have suggested defects in *ARSK* may lead to a new MPS subtype, MPS X, in humans. Here we report a 13-year-old cognitively intact boy diagnosed with Perthes disease and pectus carinatum referred for possible skeletal dysplasia. Exome sequencing analysis of his peripheral blood sample revealed a novel homozygous missense variant, c.1067C>A (p.S356Y) in the *ARSK* gene, functionally proven to result in ARSK deficiency, evidenced by failure to remove the 2-O-sulfate group from 2-sulfoglucuronate. Urine glycosaminoglycan (GAG) examination with a more sensitive liquid chromatography-tandem mass spectrometry (LC-MS/MS) method confirmed a significant increase in dermatan sulfate, suggesting malfunction of glycosaminoglycan metabolism. Skeletal abnormalities of vertical striae of distal femoral bones and bilateral Perthes were consistent with those observed in previous cases.^1^^,^^2^ Based upon the collective results of functional studies, urine GAG analysis, and skeletal findings, the novel homozygous missense germline variant in *ARSK* (c.1067C>A, p.S356Y) was determined to cause an MPS disorder due to ARSK deficiency. This novel *ARSK* pathogenic variant will be useful in diagnosis/prognosis and highlights the important and unique roles of ARSK in the degradation of sulfated glycosaminoglycans and maintenance of normal lysosomal functions in humans.

A 13-year-old cognitively intact boy of Syrian ancestry was diagnosed with bilateral Perthes disease and pectus carinatum. He was referred for possible skeletal dysplasia. His hip problem became evident when he was 11 years old. He has muscle pain, no fractures and can only walk a short distance due to pain, and has a pronounced limp. He was born to healthy parents who denied consanguinity. His birth weight is about 7 lbs (birth length is unavailable). He had a normal early development, met all milestones, and attended regular classes in schools. His height is at the 18th percentile, relatively short for his mean parental height (father: 5 feet 10 inches; mother: 5 feet 3 inches). His arm span-to-height ratio is 1.06; upper to lower segment ratio is 0.85. His hand was 18.8 cm (90th %ile) and middle finger 7.5 cm (50th–75th %ile). He does not have syndactyly or camptodactyly. His right and left calves were 34 cm and 33.5 cm in circumference respectively. He has widely spaced teeth and thickened alveolar ridges. However, he does not have a strikingly coarse facial appearance (Fig. 1A, B). Ophthalmology evaluation showed exophoria without a pattern, normal optic nerves, and no lens or vitreous opacity. He has minimal hyperopic astigmatism with excellent vision and normal dilated examination with prominent optic nerves without pallor and no retinal pigmentation changes.

**Figure 1:**
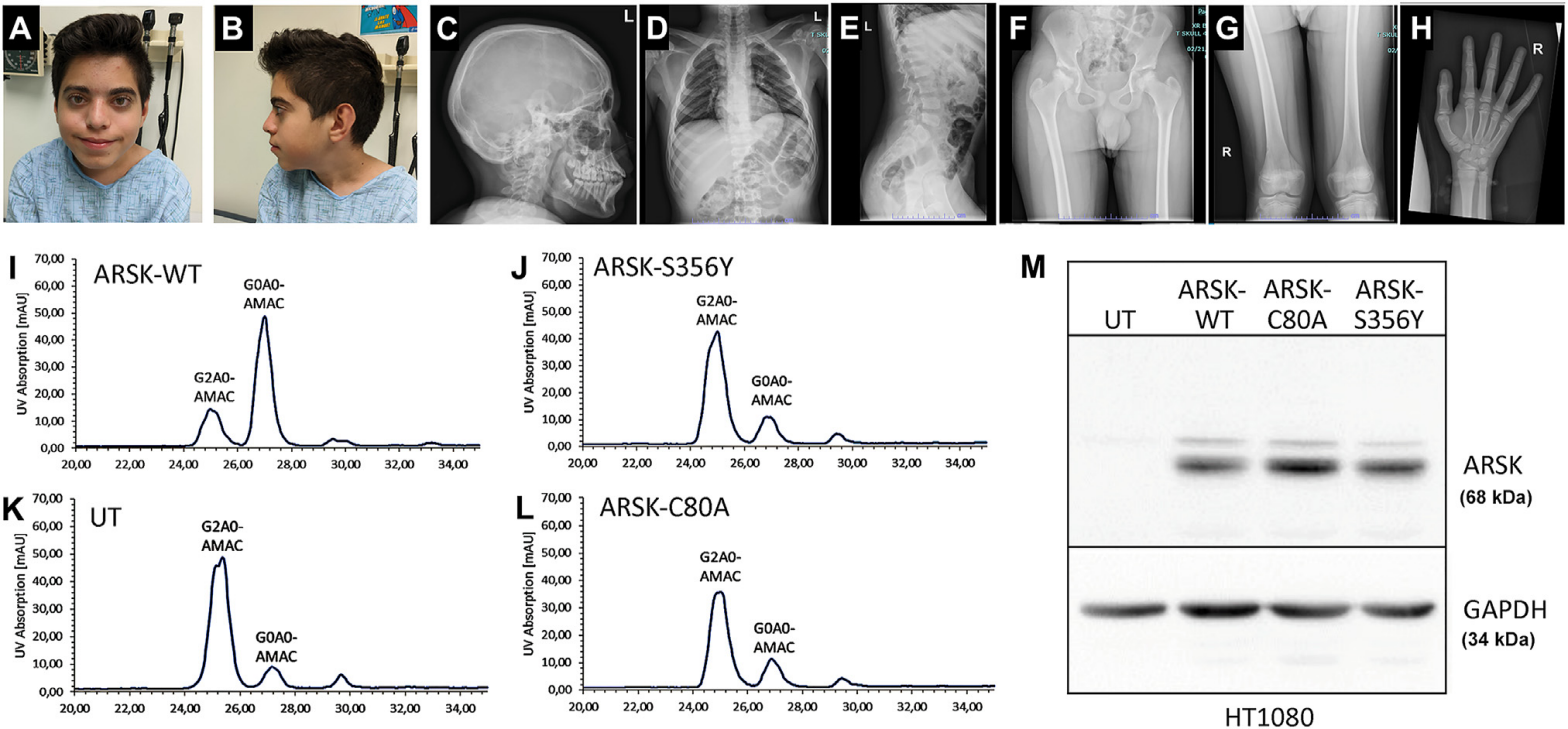
X-ray survey of the patient and functional consequence of the *ARSK* variant (p.S356Y). **(A, B)** Facial view: no obvious or very mild coarse facial appearance. **(C–H)** X-ray survey of the skull, chest, spine, hip, femurs, femora heads, carpal bones, and metacarpals. **(I–M)** ARSK wild-type (ARSK-WT) but not the mutant (ARSK-S356Y) desulfates synthetic 2-sulfoglucuronate-N-acetylglucosamine (G2A0) disaccharides. (I) AMAC-labelled G2A0 disaccharide treatment with cell lysates expressing ARSK-WT resulted in a minor peak at 25 mL retention volume representing the 2-O-sulfated educt and a major AMAC peak at around 27 mL representing the desulfated product, indicating the loss of the sulfate group. (J–L) AMAC-labelled G2A0 disaccharide treated with cell lysates expressing the mutant ARSK-S356Y (**J**), mutant ARSK-C80A (L), or just expressing endogenous ARSK of HT1080 cells (UT) (K) resulted in a main AMAC peak at 25 mL. The G0A0-mediated fluorescence signal at 27 mL volume remained the minor peak in both samples (J, L). Of note, the 27 mL minor peak in ARSK-S356Y, ARSK-C80A, and UT most likely results from the activity of the endogenous ARSK of HT1080 cells. The ubiquitous peak on the right of the chromatogram (>30 mL of retention volume) was not analyzed in more detail as it was also present in untreated samples. Analysis in (I–L) was performed with C18-reversed-phase chromatography. (M) Western blot showed comparable expression levels for ARSK-WT, ARSK-C80A, and ARSK-S356Y in HT1080 cell lysates, which indicated comparable stabilities of ARSK-WT and ARSK-S356Y. GAPDH was used as a loading control.

His skeletal survey showed partially collapsed concave bilateral femoral capital epiphyses and bilateral incomplete fusion of the anterior inferior iliac spine, striated trabecular appearance of the ends of the long bones, slight genu valgum bilaterally, distorted talar domes with medial talar tilt, platyspondyly with anterior beaking, exaggerated thoracic kyphosis, minor pectus carinatum, slight brachycephaly, and normal bone densities, consistent with skeletal dysplasia, “likely spondyloepiphyseal dysplasia” (Fig. 1C, E–H). There is a small fibrous cortical defect at the distal right femoral diametaphysis laterally. At the time of the examination, he had a normal cardiac silhouette with slight underinflation exaggerating the central pulmonary markings on the chest X-ray (Fig. 1D). He also had normal levels of dermatan sulfate, heparan sulfate, keratan sulfate, and chondroitin-6-sulfate in his urine quantified with a standard dimethyl methylene blue test in an academically based diagnostic laboratory (the test results are shown in Table S1). A gene panel associated with skeletal abnormalities did not reach a diagnosis.

With the exome sequencing analysis, we identified a novel homozygous missense germline variant, c.1067C>A (p.S356Y), in the *ARSK* gene (reference sequence: NM_198150.3) in the patient. Both parents were confirmed as heterozygous carriers (Fig. S1). This variant occurs at a highly conserved amino acid position within the conserved sulfatase domain. *In silico* predictions suggest a deleterious effect of this change. This variant has not been reported either as a benign or disease-causing variant in humans. It has not been observed in the gnomAD database, the largest population allele frequency database (https://gnomad.broadinstitute.org/).

Arylsulfatase K (ARSK) is a recently characterized lysosomal hydrolase involved in GAG degradation that removes the 2-O-sulfate group from 2-sulfoglucuronate.^3^ Glucuronate-2-O-sulfation occurs in heparan sulfate, dermatan sulfate, and chondroitin sulfate and is selectively removed by ARSK during degradation.^3^ Knockout of *Arsk* in mice was consistent with mild storage pathology.^4^ To verify the functional consequence of the *ARSK* alteration due to the variant of p.S356Y identified in our patient, the enzyme activity of this ARSK mutant was assessed using a specific *in vitro* system (see details in Supplemental Methods). In brief, ARSK wild-type (ARSK-WT), a known loss-of-function ARSK mutant (ARSK-C80A), and the mutant construct containing the variant p.S356Y (ARSK-S356Y) were generated via site-directed mutagenesis. A specific ARSK substrate, 2-O-sulfoglucuronate-containing disaccharides, G2A0, which was prelabelled with the fluorescent dye AMAC at the reducing end of the N-acetylglucosamine residue,^1^ was used to measure the enzyme activities. After expressing ARSK-WT, ARSK-C80A, and ARSK-S356Y constructs in HT1080 cells respectively, the cell lysates were incubated with AMAC-labelled G2A0, which were subsequently analyzed with C18-reversed-phase chromatography. As shown in Figure 1I–M, similar to ARSK-C80A, ARSK-S356Y completely abolished the ARSK enzyme activity compared with the wild-type (ARSK-WT) (Fig. 1I–L), while the mutants had a comparable level of protein expression and stability with the wild type (Fig. 1M). These results indicate that ARSK-S356Y loses the ability to desulfate glucuronate-2-O-sulfated disaccharide efficiently, as does ARSK-C80A, a known loss-of-function variant,^1^^,^^3^ suggesting our patient may likely have abnormal ARSK sulfatase.

To verify the *ARSK* variant (p.S356Y) has a negative effect on GAG degradation in our patient, we determined to re-evaluate his urine GAG excretion with an alternative more sensitive method of the enzymatic digestion followed by liquid chromatography-tandem mass spectrometry (LC-MS/MS).^1^^,^^5^ The repeat urine GAG examination showed a significant increase in the dermatan sulfate (247 ug/mmol, reference values: 0–53 ug/mmol creatinine; Table S2), though heparan sulfate and keratan sulfate levels remained within normal ranges (Table S2). GAG levels in his plasma were not evaluated as these values were not shown abnormal in previously reported patients.^1^ This result is consistent with ARSK deficiency due to the variant p.S356Y demonstrated by the functional assessment (Fig. 1J), suggesting that the patient is affected with MPS disorder due to ARSK deficiency.

To date, only three *ARSK* variants (p.R84C, p.L184X, p.Y417X) in six individuals from three unrelated families have been described in the literature, in whom the new MPS subtype due to ARSK deficiency, MPS X, was suggested.^1^^,^^2^ To our knowledge, this is the fourth variant (p.S356Y) and the seventh patient reported to date with this new MPS subtype. Clinical findings, phenotypical features, and *ARSK* variations for these seven individuals are summarized in Table S3. By searching the gnomAD database (https://gnomad.broadinstitute.org/), we found approximately 386 unique missense, in-frame and truncated variants in the *ARSK* gene observed in general populations. More than 95% (370/386) of them have been observed with an allele frequency of less than 1 in 10,000, and only 7 variants have homozygotes. Given this observed allele distribution, frequency, and inheritance, the incidence of *ARSK*-related MPS disorders would expect to be low, which is consistent with the reported incidence in the literature.

All affected individuals reported are childhood onset with relatively mild MPS features, such as a mild elevation of dermatan sulfate and lack of cognitive effects, as observed in our patient. Additional cardiac and ophthalmological abnormalities were observed in some on follow-up examination.^1^ As with previously reported individuals,^1^^,^^2^ the urine GAG levels measured by a standard dimethyl methylene blue test in our patient at the initial evaluation were within normal ranges (Table S1, 3). However, given the difficulty in recognizing this MPS subtype due to its similarity to some types of skeletal dysplasias, such as spondyloepiphyseal dysplasia, and because of its atypical MPS features, it is possible that this condition may be underdiagnosed. Further evaluation with the more sensitive diagnostic method for urine GAG levels should be considered. Molecular genetic testing followed by functional assessment if necessary is very important and helpful for defining disease etiology.

In conclusion, our exome findings of the homozygous *ARKS* variant (p.S356Y) combined with the constellation of clinical presentation suggest that our patient has the newly defined MPS type X condition. The complete loss of ARSK enzyme activity due to the p.S356Y variation was demonstrated by an ARSK-specific enzymatic assay. Repeat urine MPS testing with an alternative method of LC-MS/MS analysis confirmed the GAG storage defect in our patient. Based on these findings, one would expect that the urinary dermatan sulfate should be enriched in non-reducing end 2-sulfo-glucuronic acid in the patient and should be evaluated in future follow-ups. Continued evaluations will provide more a comprehensive picture of the *ARSK*-related disease course, and heightened awareness should lead to the diagnosis of other affected individuals. We have referred him to Cardiology, Orthopedics, and the Pain Clinic. We will further evaluate his pulmonary function. It will be very important to develop enzyme replacement therapy and potentially other treatments, given the pain and disability associated with this disorder.

## Author contributions

M.S., T.L., and L.M.R. conceived and designed the project. M.S., C.K.K., F.M.A., B.P., T.L., P.D., M.I., and L.M.R. conducted experiments and/or data analysis. M.S. and L.M.R. provided case reports for the patients. M.S. and L.M.R. wrote the manuscript and all the authors provided feedback.

## Conflict of interests

The authors declare no conflict of interests.
