# Supplementary material for: A novel homozygous missense variant in ARSK causes MPS X, a new subtype of mucopolysaccharidosis
Source: Genes Dis. 2023 Jul 10;11(3):101025. doi: 10.1016/j.gendis.2023.06.003 (PMC10825285; doi:10.1016/j.gendis.2023.06.003)
Supplement: Multimedia component 1 [file mmc1.docx]

**Supplementary Data**

**A Novel Homozygous Missense Variant in *ARSK* Causes MPS X, A New Subtype of Mucopolysaccharidosis**

Miao Sun^1*^, Cornelia K. Kaminsky^2^, Philip Deppe^3^, Mai-Britt Ilse^3^, Frédéric M. Vaz^4,5,6^, Barbara Plecko^7^, Torben Lübke^3^, Linda M. Randolph^8**^

^1^Division of Genomic Medicine, Department of Pathology and Laboratory Medicine, Children's Hospital Los Angeles/Keck School of Medicine of USC, Los Angeles, CA, 90027, USA

^2^Department of Radiology, Children's Hospital Los Angeles/Keck School of Medicine of USC, Los Angeles, CA, 90027, USA

^3^Department of Chemistry, Biochemistry, Bielefeld University, 33615 Bielefeld, Germany

^4^Amsterdam UMC location University of Amsterdam, Department of Clinical Chemistry and Pediatrics, Laboratory Genetic Metabolic Diseases, Emma Children's Hospital, Meibergdreef 9, 1100 DE Amsterdam, The Netherlands

^5^Amsterdam Gastroenterology Endocrinology Metabolism, Inborn errors of metabolism, 1105 BK Amsterdam, The Netherlands

^6^Core Facility Metabolomics, Amsterdam UMC location University of Amsterdam, 1100 DD Amsterdam, The Netherlands

^7^Department of Pediatrics, Division of General Pediatrics, Medical University of Graz, 8036 Graz, Austria

^8^Division of Medical Genetics, Department of Pediatrics, Children's Hospital Los Angeles/Keck School of Medicine of USC, Los Angeles, CA, 90027, USA

^*^Corresponding author: miaosun@chla.usc.edu (M.S.)

^**^Senior author: LRandolph@chla.usc.edu (L.M.R.)

**Supplemental Tables**

**Table S1.** Results of DMB* test of GAG** excretion in urine

| **Mucopolysaccharides Quant Urine** | **Result** | **Reference values** |
| --- | --- | --- |
| Dermatan sulphate | 0.16 | < 1.0 |
| Heparan sulphate | 0.09 | < 0.25 |
| Chondroitin-6 Sulfate | 0.90 | <= 1.50 |
| Keratan sulphate | 0.20 | < 0.5 |

*DMB dimethyl methylene blue; **GAG glycosaminoglycans

**Table S2.** Results of the enzymatic digestion followed by liquid chromatography-tandem mass spectrometry (LC-MS/MS) analysis of urine GAGs

| **Glycosaminoglycans (GAGs)** | **ug/l** | **ug/mmol creatinine (Reference values)** |
| --- | --- | --- |
| Dermatan sulphate | 3385 | 247 (0 – 53) |
| Heparan sulphate | 3838 | 280 (0 – 323) |
| Keratan sulphate | 3943 | 288 (0 – 314) |

**Table S3.** Summary of phenotype and genetic variants of individuals with ARSK deficiency

|  | **Patient in this study** | **Subject 1**  **(family 1) ^b^** | **Subject 2**  **(family 1) ^b^** | **Subject 3**  **(family 2) ^b^** | **Subject 4**  **(family 2) ^b^** | **Individual 1 ^c^** | **Individual 2 ^c^** |
| --- | --- | --- | --- | --- | --- | --- | --- |
| Variant in the *ARSK* ^a^ | c.1067C>A (p.S356Y) homozygous | c.250C>T (p.R84C) homozygous | c.250C>T (p.R84C) homozygous | c.560T>A (p.L187X) homozygous | c.560T>A (p.L187X) homozygous | c.1251C>G (p.Y417X) homozygous | c.1251C>G (p.Y417X) homozygous |
| Ethnicity | Syrian | Turkish | Turkish | Indian | Indian | Norwegian | Norwegian |
| Age in years, gender (at examination) | 13 years, male | 16 years, female | 14 years, male | 18 years, male | 17 years, male | 9.4 years, male | 11 years, female |
| Birth weight | 7 lbs | NA | NA | NA | NA | 2610 g, <1st %ile | 3014 g, 9th %ile |
| Birth length | NA | 50th %ile | 50th %ile | NA | NA | 46 cm, 1st %ile | 50 cm, 42nd %ile |
| Birth head circumference | NA | Macrocephaly | Macrocephaly | NA | NA | 34 cm, 18th %ile | 34 cm, 17th %ile |
| Previous suspected diagnoses | Spondyloepiphyseal dysplasia, Perthes disease, MPS | MPS, spondyloepiphyseal dysplasia, Turner syndrome | MPS, spondyloepiphyseal dysplasia | Brachyolmia, MPS, spondyloepiphyseal dysplasia | Brachyolmia, MPS, spondyloepiphyseal dysplasia | LCPD, MED, Meyers dysplasia MPS IV | MPS IV |
| Height | 150.4 cm, 18th %ile | 146.5 cm, -3.25 SD | 150 cm, -1.94 SD | 157 cm, -3.22 SD | 145.5 cm, -4.53 SD | 149.5 cm at age 9.4 years, SDS 1.93 | 149.5 cm at age 11 years, SDS 0.40 |
| Weight | 48 kg, 55th %ile | 68 kg | 49 kg | NA | 32.48 kg | 64 kg, 17 kg above 97.5th %ile | 37.1 kg, 50th %ile |
| Occipitofrontal circumference | 54 cm, 42nd %ile | 59 cm, 2.52 SD | 58.5 cm, 1.95 SD | 53 cm, -2.21 SD | 52.5 cm, -2.41 SD | 55 cm, SDS 1.17 | 54 cm, SDS 0.64 at age 10.8 years |
| Arm span | 159 cm | 150.5 cm | 155 cm | 164 cm | 147 cm | 151 cm | 148 cm |
| Length ratio of upper/lower body segment | 0.85 | 0.95 | 0.88 | 0.87 | 0.82 | NA | NA |
| Facial phenotype | Not strikingly coarse facial appearance | Coarse facial features | Coarse facial features | Coarse facial features, long philtrum, broad nasal root | Coarse facial features, long philtrum, broad nasal root | Not coarse facial features | Malar hypoplasia, not coarse facial features |
| **Table S3.** (Continue) | | | | | | | |
| Eye examination | Eyes are prominent, exophoria without pattern, normal optic nerves and no lens or vitreous opacity, minimal hyperopic astigmatism with excellent vision, normal dilated examination with prominent optic nerves without pallor, no retinal pigmentation changes | Mild myopia since 14th year of life, mild lens and vitreous opacity, mild retinal pigmentation temporal of the fovea | Mild lens and vitreous opacity, mild retinal pigmentation temporal of the fovea | Normal | Normal, no corneal opacity, normal fundus | Normal | Slight myopia at age 11.3 years |
| Auditory system | Normal | Normal audiogram and tympanogram | Normal audiogram and tympanogram | Normal | Normal | Normal audiogram | Normal audiogram |
| Jaw and teeth | Widely spaced teeth, thickened alveolar ridges | Open bite, wide spaced teeth, diastemata, canine‐like appearance of lateral incisors | Open bite, wide spaced teeth, canine‐like appearance of lateral incisors | Normal | Normal | Slightly hypomineralized enamel | Slightly hypomineralized enamel |
| Hands/wrists | Normal | Normal | Normal, intermittent paresthesias | Normal | Brachydactyly, arthropathy of right wrist | Hypermobile distal interphalangeal joints and first metacarpophalangeal joint | Hypermobile distal interphalangeal joints and first metacarpophalangeal joint |
| Skeletal features | Relatively short‐trunk short stature, slightly genu valgus, vertical striae of distal femoral bones, bilateral Perthes | Disproportionate short‐trunk short stature, genu valgus, mild scoliosis | Disproportionate short‐trunk short stature, mild genu valgus | Disproportionate short‐trunk short stature, genu valgus | Disproportionate short‐trunk short stature, genu valgus, mild scoliosis | Proportionate osteochondral lesions | Proportionate osteochondral lesions |
| Liver, spleen | No hepatosplenomegaly on clinical examination | Normal in size and structure (ultrasound examination) | Normal in size and structure (ultrasound examination) | Normal on clinical examination | Normal on clinical examination | Slightly hyperechogenic liver, otherwise normal (ultrasound) | Normal (ultrasound) |
| **Table S3**. (Continue) | | | | | | | |
| Kidneys | NA | Normal in size and structure (ultrasound examination) | Normal in size and structure (ultrasound examination) | NA | Renal calculus at 6 years | Normal (ultrasound) | Normal (ultrasound) |
| Heart | Normal cardiac silhouette with slight underinflation | Systolic murmur, mild aortic valve stenosis and regurgitation, thickened ends of aortic cusps, mild left ventricular hyperthrophy | Systolic and diastolic murmur, mild aortic valve stenosis and regurgitation, thickened ends of aortic cusps | Normal on clinical examination | Normal on clinical examination | Normal ECG, possible slight diastolic dysfunction, otherwise normal echocardiogram | Normal ECG and echocardiogram |
| Neurological examination, cognition | Normal | Normal | Normal | Normal | Normal | Normal, possible Tourette syndrome | Normal |
| Urinary GAG (DMB test) | Normal at 13 years | Normal at 11 and 16 years | Borderline at 14 years | NA | NA | At age 8 years, U‐GAG 13 mg/mmol creatinine (age-adjusted reference range: 5–11); at age 9 years, U‐GAG 14 mg/mmol creatinine (age‐adjusted reference range: 5–11) | At age 10 years, U‐GAG 14 mg/mmol creatinine (age‐adjusted reference range: 4–11); U‐GAG 15 mg/mmol creatinine on repeat analysis (age‐adjusted reference range: 4–11) |
| LC–MS/MS Metabolic diseases ^d^ | DS 247 μg/mmol (reference values: 0–53 μg/mmol creatinine) at age 13 years | DS 165 μg/mmol (reference values: 0–53 μg/mmol creatinine) at age 17 years | DS 234 μg/mmol (reference values: 0–53 μg/mmol creatinine) at age 15 years | NA | NA | DS 338 μg/mmol creatinine (reference values: 0–109 μg/mmol creatinine) at 9.4 years | DS 358 μg/mmol (reference values: 0–53 μg/mmol creatinine), HS 365 μg/mmol creatinine (reference values: 0–323 μg/mmol creatinine) at age 10.9 years |

^a^ Reference sequence: NM_198150.3; ^b^ Verheyen et al., J Med Genet, 2022, 59:957-964; ^c^ Rustad et al., Am J Med Genet, 2022, 188A:3369-3373; ^d^ Analysis performed at Amsterdam Universitair Medische Centra, Academic Medical Center, Lab Genetic Metabolic diseases; NA, not available; ARSK, arylsulfatase K; MPS, mucopolysaccharidosis; LCPD, Legg-Calvè-Perthes disease; MED, multiple epiphyseal dysplasia; DS, dermatan sulfate; HS, heparan sulfate.

**Supplemental Figures**

**
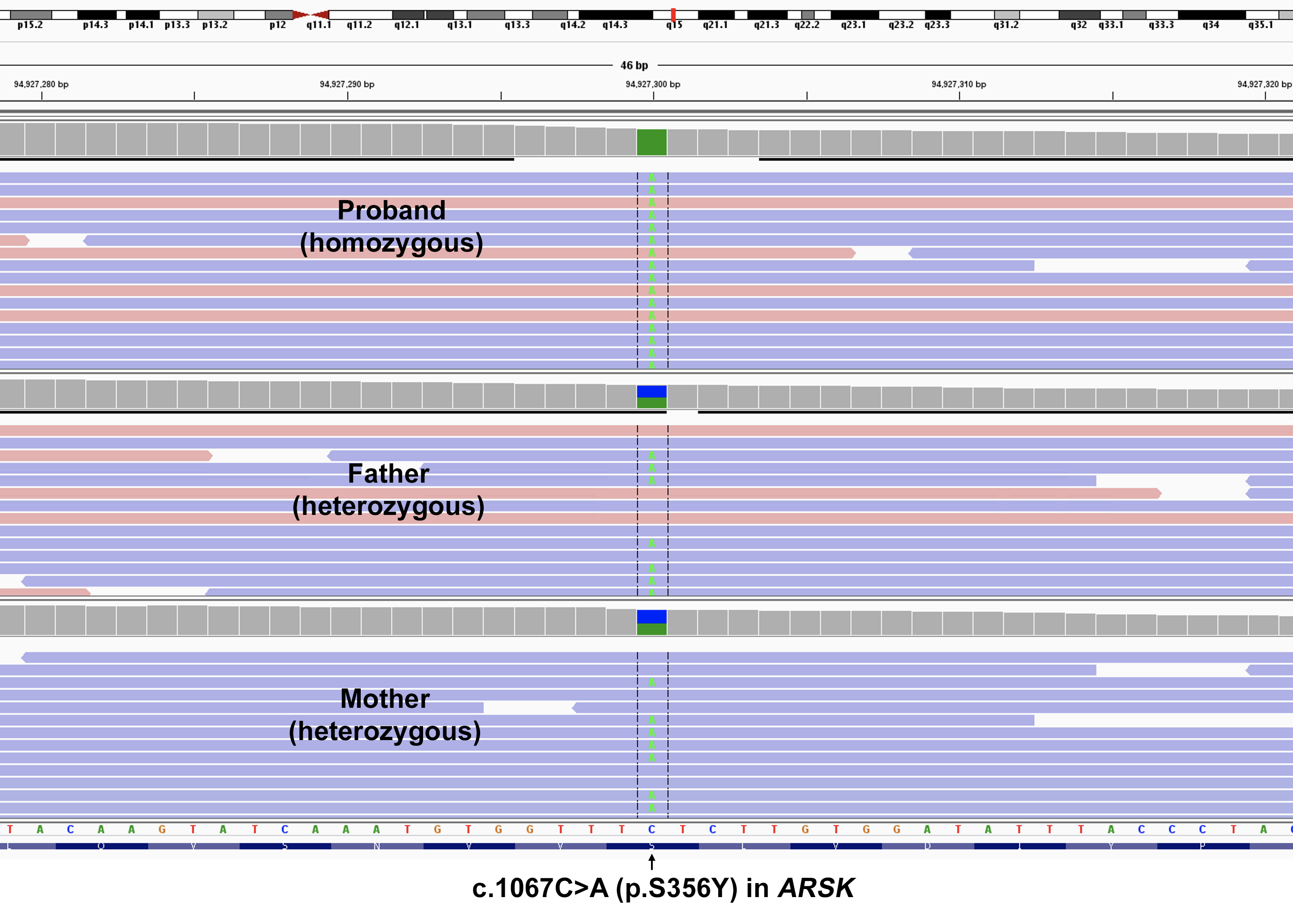
**

**Figure S1.**  IGV picture showing the exome sequencing-identified variant in the *ARSK* gene locus in chromosome 5q15 region. Top panel is from the patient and the bottom two panels are from the patient’s parents.

**Supplemental Materials and Methods**

**Exome sequencing and analysis**

Genomic DNA was extracted from the peripheral blood of the proband and parents using a commercial kit - Promega Maxwell RSC DNA Extraction Kit (Madison, WI, USA). The Exome Sequencing library was generated using the Agilent SureSelect Human All Exon V6 plus a custom mitochondrial genome capture kit. Captured DNA fragments were then sequenced using the Illumina Nextseq 500 or HiSeq 4000 sequencing system, with 2x100 base-pair (bp) paired-end reads. Single nucleotide variants, and small insertions and deletions (<10 bp) were detected by mapping and comparing the DNA sequences with the human reference genome (GRCh37/hg19). To identify the potential disease-causing variants, a primary gene list was generated based on the phenotype-related keywords provided by the physician and thereafter standardized to human phenotype ontology terms, using the phenotype-gene-disease correlation database from the Human Phenotype Consortium (Released in February, 2021) for prioritization of variant analysis and interpretation. The rare nuclear DNA variants (minor allele frequency <1%) within protein-coding regions and splice-site junctions (5 bp into introns) and any rare mitochondrial DNA variants with <0.5% MitoMap GB frequency were further annotated and analyzed using a commercial tool Agilent Alissa Interpret 5.2 (Santa Clara, CA, USA). Sequence variant classification and interpretation is based on ACMG/AMP standards and guidelines ^1^. Variant confirmation by Sanger sequencing was performed for all insertions and deletions as well as substitutions that did not meet the coverage and quality score thresholds (Score < 500 and read depth < 10).

**Laboratory investigations**

GAG excretion in urine (24-hour collection and random sample) was analyzed quantitatively with a standard DMB test at Mayo Clinical Laboratories (MN, USA). Urine GAGs were also measured using a multiplex assay with enzymatic digestion of HS, DS and KS followed by quantification of specific disaccharides by LC-MS/MS as described previously ^2^.

**Generation of ARSK constructs**

ARSK wild-type (ARSK-WT) and ARSK-C80A (positive control) constructs that were used for comparison of enzymatic function and specificity were constructed as described previously ^3^. ARSK mutant (ARSK-S356Y) construct was generated to prove that the detected variant, NM_198150.3:c.1067C>A (p.S356Y), results in reduced enzymatic function with a specific enzyme assay. The ARSK-S356Y construct was derived by site-directed mutagenesis according to the QuickChange mutagenesis protocol (Agilent Technologies) using the following mutagenesis primers:

ARSK_c.1067C>A_f: CAAGTATCAAATGTGGTTT**A**TCTTGTGGATATTTACC

ARSK_c.1067C>A_r: GGTAAATATCCACAAGA**T**AAACCACATTTGATACTTG

The sequences of the resulting constructs were validated by Sanger sequencing (data not shown).

**Transfection and immunoblotting**

ARSK-WT, ARSK-S356Y, and ARSK-C80A were transiently expressed in HT1080 cells by polyethylenimine (PEI) transfection protocol as described previously ^3^. Briefly, cells were harvested 48 hours after transfection and lysed in phosphate-buffered saline (PBS)/0.5% TX100; sonication was performed on ice (3×10 s), and homogenates were obtained by centrifugation (15000 g, 4°C). Protein determination was performed by detergent compatible (DC) assay (BioRad). Homogenates (50 μg of total protein) were analyzed by immunoblotting on polyvinylidene difluoride (PVDF) membrane and antibodies directed against ARSK (biorbyt 160024) and GAPDH (Santa Cruz sc-25778, FL-335) as loading control.

**Glucuronate-2-sulfatase activity assay**

The disaccharide 2-sulfoglucuronate-N-acetyl-glucosamine (G2A0) was pre-labelled with the fluorescent dye 2-aminoacridone (AMAC) ^3^. AMAC-labelled G2A0 (12.5 nmol) was incubated with 100 μg protein of the appropriate homogenates in a final volume of 52.5 μl in 250 mM ammonium-acetate buffer pH 4.6 for 24 hours at 37°C. After centrifugation (15000 g, 4°C), the samples were analyzed by C18-reversed-phase (RP)-chromatography in ammonium acetate buffer (60 mM, pH 5.6) with a flow rate of 1 mL/min with the Ettan LC system (GE Healthcare). The saccharides were eluted and fractionated with an acetonitrile gradient, in which AMAC-labelled disaccharides were detected by ultraviolet (UV) absorbance at 255 nm.

**REFERENCES**

1. Richards, S., Aziz, N., Bale, S., Bick, D., Das, S., Gastier-Foster, J., Grody, W.W., Hegde, M., Lyon, E., Spector, E., et al. (2015). Standards and guidelines for the interpretation of sequence variants: a joint consensus recommendation of the American College of Medical Genetics and Genomics and the Association for Molecular Pathology. Genet Med 17, 405-424.

2. Langereis, E.J., Wagemans, T., Kulik, W., Lefeber, D.J., van Lenthe, H., Oussoren, E., van der Ploeg, A.T., Ruijter, G.J., Wevers, R.A., Wijburg, F.A., et al. (2015). A Multiplex Assay for the Diagnosis of Mucopolysaccharidoses and Mucolipidoses. PLoS One 10, e0138622.

3. Verheyen, S., Blatterer, J., Speicher, M.R., Bhavani, G.S., Boons, G.J., Ilse, M.B., Andrae, D., Spross, J., Vaz, F.M., Kircher, S.G., et al. (2022). Novel subtype of mucopolysaccharidosis caused by arylsulfatase K (ARSK) deficiency. J Med Genet 59, 957-964.
